# The association of glycemic level and prevalence of tuberculosis: a meta-analysis

**DOI:** 10.1186/s12902-021-00779-6

**Published:** 2021-06-16

**Authors:** Zhifei Chen, Qi Liu, Ranran Song, Wenxin Zhang, Tingping Wang, Zhan Lian, Xuezhi Sun, Yanli Liu

**Affiliations:** 1 Wuhan Pulmonary Hospital, Wuhan, Hubei China; 2 Key Laboratory of Environment and Health, Ministry of Education and Ministry of Environmental Protection, and State Key Laboratory of Environmental Health, School of Public Health, Tongji Medical College, Huazhong University of Science and Technology, Wuhan, Hubei China; 3 Department of Maternal and Child Health and MOE Key Lab of Environment and Health, School of Public Health, Tongji Medical College, Huazhong University of Science and Technology, No 13 Hangkong Road, Wuhan, Hubei China

**Keywords:** Tuberculosis, Diabetes, Glycemic control, Meta-analysis

## Abstract

**Background:**

Diabetes is a well-known risk factor for tuberculosis and poorly glycemic control may increase the risk of tuberculosis. We performed a meta-analysis to explore the association of glycemic control in diabetic patients and their tuberculosis prevalence.

**Methods:**

We included observational studies that investigated the prevalence of tuberculosis associated with glycemic control. The markers of glycated hemoglobin A1c (HbA1c) and fasting plasma glucose were used to evaluate the exposure of interest in the study. We searched related articles in PubMed, EMBASE and Web of Science through 14 December 2019. The Newcastle-Ottawa scale was used to assess the risk of bias of included studies.

**Results:**

Seventeen studies (four cohort studies, five case-control studies and eight cross-sectional studies) were included, involving 1,027,074 participants. The meta-analysis found the pooled odds ratio of prevalent tuberculosis increased a 2.05-fold (95%CI: 1.65, 2.55) for the patients with HbA1c ≥7.0% compared to those with HbA1c concentration < 7.0%. Furthermore, we found the mean of HbA1c was higher in the diabetes mellitus with tuberculosis group than the diabetes-only group (*P* = 0.002). In the sensitivity analysis, the finding remains consistent.

**Conclusion:**

Our study provides the evidence that poorly controlled diabetes in diabetics may be associated with increased prevalence of tuberculosis. More efforts should focus on screening tuberculosis in uncontrolled diabetes.

**Supplementary Information:**

The online version contains supplementary material available at 10.1186/s12902-021-00779-6.

## Background

Tuberculosis (TB) remains one of the most common infectious diseases in the world. The World Health Organization (WHO) has published that an estimated of approximately 10.0 million people fell ill with TB in 2019 from 202 countries and territories, suggesting a high burden of TB [1]. It is necessary to identify potential high-risk factors for TB screening. Besides HIV infection, poverty, undernutrition, and smoking, diabetes mellitus (DM) has received recent recognition as a risk factor for TB [2].

Epidemiological studies have elucidated the relationship of DM with the prevalence of TB disease [3–5]. In a recent systematic review among 13 observational studies, people with DM had about tripled the prevalence of developing TB than people without DM [3]. Additionally, in the two nationwide population-based studies, there was a higher underlying prevalence of tuberculosis infection at baseline in diabetics patients compared to the healthy population [4, 5]. One explanation for these results is that dysglycemia in diabetic patients may impair their innate immune system that seems to provide a favorable environment for acute intracellular bacterial infections in diabetic patients [6, 7]. Furthermore, the intracellular bacterial infections (e.g. TB) are one of the common complications of DM [8, 9]. Dysglycemia may be a key factor that impact the relationship of DM and TB.

A great number of studies have focused on the role of dysglycemia on the prevalence of TB [10–14]. Patients with poorly controlled diabetic are related to an increased prevalence of developing TB compared to those with controlled blood glucose [10–12]. In a cohort study of older individuals in China, DM subjects with baseline glycated hemoglobin A1c (HbA1c) ≥7% had higher prevalences of developing active pulmonary TB than patients with HbA1c < 7% (adjusted hazard ratio [HR]: 3.11) [10]. Similarly, Lin et al. showed that diabetics patients with poor glucose control were especially vulnerable to developing TB compared to the controlled DM [12]. However, there are inconsistent findings [13, 14]. The cohort study in U.K. by Pealing et al. indicated that patients with poorly controlled DM did not increase the prevalence of TB compared with diabetic patients with controlled glucose [13]. A case-control study in Denmark also reported that the level of HbA1c was not significantly related to the prevalence of TB [14]. Therefore, whether glycemic control mediates the relationship between DM and TB infection is unknown.

Considering the increasing prevalence of DM over time and greater susceptibility to infections [15], in the present study, we conducted a meta-analysis to examine the association between glycemic level and TB prevalence among patients with diabetes.

## Methods

### Search strategy and eligibility criteria

In the present study, we searched PubMed, Embase, and Web of Science to identify all relevant articles exploring the association of poor glycemic control and TB prevalence before December 14, 2019, without language restrictions. We searched the main keywords included “diabetes mellitus”, “DM”, “Glycated Hemoglobin A”, “hemoglobin A1c”, “HbA1c”, “fasting plasma glucose”, “FPG”, “glycemic control”, “Tuberculosis”, “TB”, and “mycobacterium”. Medical subject headings (MeSH) terms were used to efficiently search PubMed, and similar key words were used to search Embase and Web of Science. The search strategy for each database is available in the Additional file (Additional file 1).

**Fig. 1:**
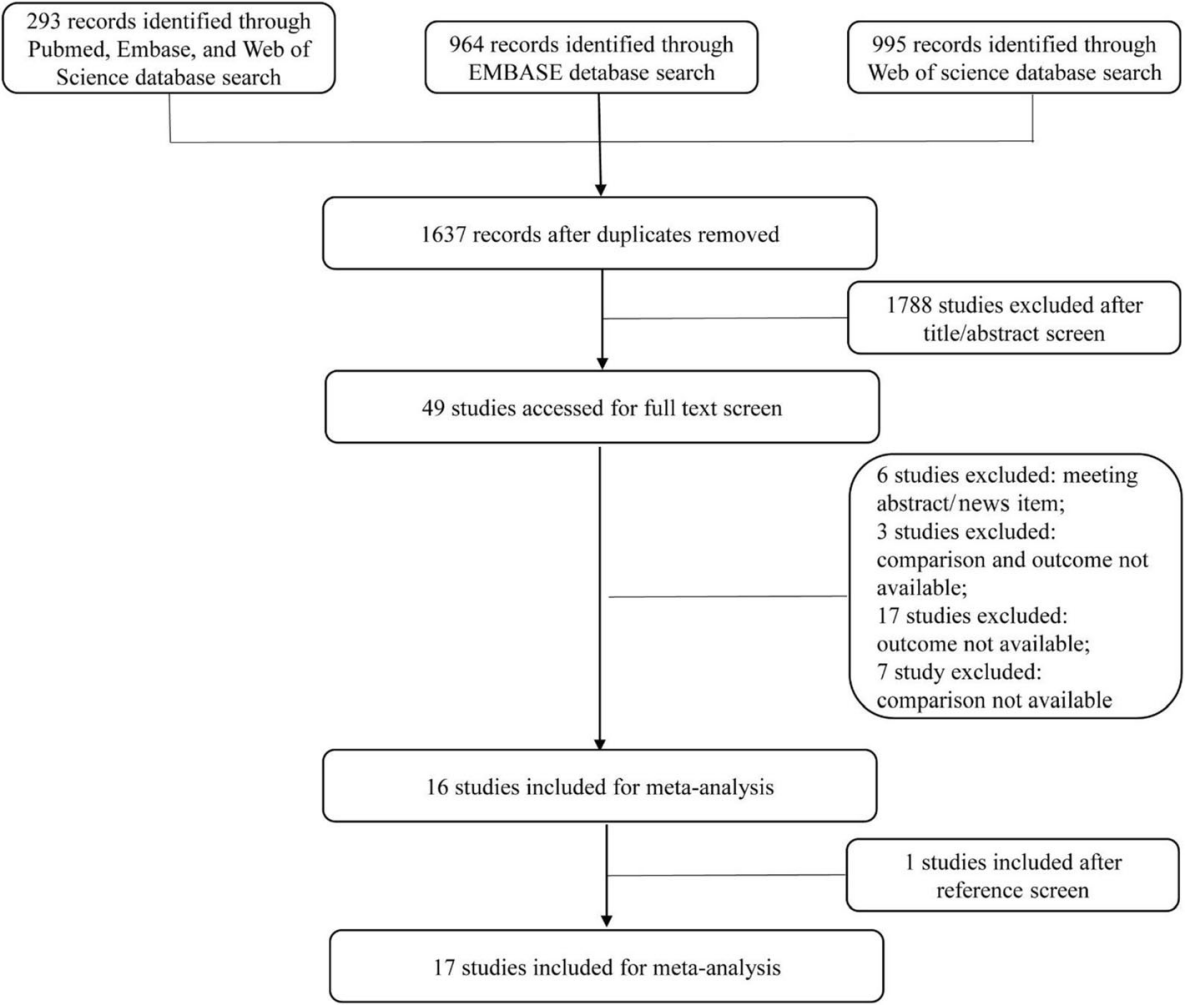
Flow chart of literature search

To include as many studies as possible, searches were not restricted by publication date or study type. We included articles that met the following three inclusion criteria: published up to December 14, 2019, published in English; included exposure of interest which evaluated glycaemia control status in diabetes patients; included outcome of interest which investigated the prevalence of pulmonary tuberculosis. We included the study that restricted to patients with diabetes only. Case reports, meeting abstracts, news items, articles without available comparison and outcome, articles not published in English, and articles exploring extrapulmonary tuberculosis were excluded. To evaluate the selection process, the study selection flowchart outlines records at each step (Fig. 1).

### Data extraction and analysis

The value of HbA1c was used as a predictor for monitoring glycemic control in DM patients. Poor glycemic control was defined as HbA1c > 7% [16]. In addition, fasting plasma glucose (FPG) was also used as an interesting marker for reflecting impaired glucose regulation under basal conditions. We used a preconceived and standardized data extraction form to collect basic information on the first author’s name, year of publication, country, the recruitment period, study design, number of participants, outcomes of studies. The reported estimated effect (e.g., Relative Risk [RR], Odds Ratio [OR], Hazard Ratio [HR]) of the association between TB prevalence and poor diabetes control was obtained from the study. As counting data, we extracted the frequency of TB patients in each exposure group to calculate the odds ratio (or) and 95% confidence interval (CI). We also extracted the mean with standard deviation (SD) or median with inter-quartile range (IQR) to examine the distribution differences in the two groups of TB-DM and DM-only groups. Relevant citations were screened by two reviewers independently and disagreements were resolved through discussion.

A meta-analysis was performed to explore the effect of blood glucose levels-on TB in DM patients. First, HbA1c is the most commonly used index for reflecting blood glucose status. We examined the relationship between HbA1c and the prevalence of pulmonary TB in DM patients: grouped by HbA1c ≥ 7% and HbA1c < 7%; Then, HbA1c was taken as a continuous variable to observe the difference of its mean value between TB-DM group and DM only group; finally, we focused on the fasting blood glucose index, and explored the mean difference between the two groups (TB-DM group and simple DM group). The observed OR of TB among persons with various glycemic control within each study was presented in forest plots. Furthermore, we observed the mean difference in the two groups (TB-DM group vs. DM-only group). Relative risk of publication bias was evaluated by visual examination of funnel plots and assessed by Egger’s test. All statistical analyses were performed with Review Manager 5.3.

The quality of included study was assessed by using the Newcastle-Ottawa Scale for case-control study and cohort study in scientific research (Table S1). Newcastle-Ottawa Scale assigns a maximum of 9 stars (4 for selection of study population, 2 for comparability, 3 for robustness of outcome or exposure).

## Results

Potentially 2252 relevant articles were identified through a comprehensive search of the databases. After removal of duplicates, 1637 articles were included. Full-text screening was performed in 49 potential eligible articles, of which 33 studies were excluded for various reasons mentioned in Fig. 1. Considering the related articles, seventeen studies (four cohort studies, five case-control studies and eight cross-sectional studies) including 1,027,074 participants met the eligibility criteria for the final review.

Baseline characteristics of the seventeen included studies are summarized in Tables 1-2. There were four cohort studies published between 2008 and 2019 in Asia. Three studies were conducted in general populations, and one was among the elderly. Study sample size of cohort studies ranged from 6444 to 819,051. Of the five case-control studies, four were conducted in high prevalence populations of developing countries. In regard of cross-sectional studies, the majority of study sample size were less than 700 (6 out of 8).

**Table 1:** Summary characteristics of 17 observational studies in the review

Study	Location	Age	Period	Populations	Outcomes
Cohort study					
Golub 2019	Korea	Adults	2001–2011	Non-DM (*n* = 766,231), DM (*n* = 52,820)	Incident TB, recurrent TB
Lee 2016	Taiwan, China	≥ 30 years	2005–2012	DM (*n* = 11,260)	Active TB
Leung 2008	China	≥ 65 years	2000–2005	DM (*n* = 6444)	Active TB
Qiu 2017	China	≥18 years	2004–2014	DM (*n* = 170,399)	TB
Case-contol study					
Khalil 2016	Egypt	All populations	2014–2015	DM (*n* = 80)	Active TB
Leal 2019	Brazil	≥18 years	2007–2013	DM (*n* = 135)	TB
Leegaard 2011	Denmark	Adults	1980–2008	Non-DM (*n* = 5578), DM (*n* = 297)	Active TB
Marupuru 2017	India	≥40 years	2015–2016	DM (*n* = 451)	TB
Widjaja 2018	Indonesia	36–86 years	2017–2017	DM (n = 80)	TB
Cross–sectional study					
Almeida-Junior 2016	Brazil	≥18 years	2010–2011	DM (n = 80)	TB
Berkowitz 2018	South Africa	Adults	2014–2015	DM (*n* = 440)	Active TB
Chan 2019	Malaysia	≥ 60 years	2016–2016	DM (*n* = 4209)	Active TB
Hensel 2016	USA	≥21 years	2013–2014	Non-DM (*n* = 406), Pre-DM (*n* = 235), DM (*n* = 54)	Latent TB
Kumpatla 2013	India	⩾15 years	2012	DM (*n* = 6967)	TB
Martinez-Aguilar 2015	Mexico	Adults	2006–2007	DM (*n* = 600)	Latent TB
Sanchez-Jimenez 2018	India	Adults	Not found	DM (*n* = 50)	Active TB
Webb 2009	South Africa	< 21 years	2006–2007	DM (*n* = 258)	Active TB

Abbreviation: DM: Diabetes mellitus, TB: Tuberculosis

**Table 2:** Summary characteristics of 17 observational studies in the review

Study	Outcomes	Inclusion of diabetic patients	Definition of TB	Parameters	Estimated OR/RR/HR (95% CI)	Adjustment
Cohort study						
Golub 2019	Incident TB, recurrent TB	DM was identified using International Classification of Diseases, Tenth Revision (ICD-10) codes E10–E10x and E14–E14x; defined using the following criteria: 1) FSG at baseline, 2) out-patient treatment for DM (at least three visits for DM care during a 365-day window) or 3) at least one hospitalization due to DM.	TB was identified using ICD-10 codes A15–A19.10; incident TB was defined by any of the following during follow-up: 1) hospitalization due to TB, 2) two or more out-patient visits for TB, or 3) receipt of at least three anti-TB medications. Prevalent TB was defined as meeting the criteria listed above either at baseline or between 1997 and 2000. Recurrent TB was considered to have occurred if the criterion for incident TB was met in a participant with previous or prevalent TB.	FSG (mean, < 5.0, 5.0–5.6, 5.6–7.0, 7.0–7.8, ≥7.8 mmol/L)	Adjusted HR: Incidence TB (Ref: FSG < 5.0 mmol/L): Male: FSG (mmol/l): 5.0–< 5.6: 0.94 (0.90–0.99); 5.6–< 7.0: 1.05 (1.00–1.11); 7.0–< 7.8: 1.50 (1.33–1.69); ≥7.8: 1.87 (1.74–2.02); Female: FSG (mmol/l): 5.0–< 5.6: 0.97 (0.90–1.03); 5.6–< 7.0: 0.97 (0.89–1.04); 7.0–< 7.8: 1.14 (0.92–1.41); ≥7.8: 1.41 (1.23–1.61); Recurrence TB (Ref: FSG < 5.0 mmol/L): Male: FSG (mmol/l): 5.0–< 5.6: 1.04 (0.97–1.12); 5.6–< 7.0: 1.11 (1.03–1.20); 7.0–< 7.8: 1.17 (0.94–1.45); ≥7.8: 2.01 (1.79–2.26); Female: FSG (mmol/l): 5.0–< 5.6: 1.04 (0.91–1.19); 5.6–< 7.0: 1.12 (0.95–1.31); 7.0–< 7.8: 1.07 (0.64–1.79); ≥7.8: 1.28 (0.91–1.80)	Adjusted for age, (age)^2^, alcohol consumption (0 g/day, < 50 g/day and ≥ 50 g/day), smoking status, BMI, (BMI)^2^, past history of cancer, past history of chronic kidney disease, medical insurance premium and (medical insurance premium)^2^.
Lee 2016	Active TB	DM status and glycemic control were defined using information from the screening service (FPG) and the national health insurance database. DM was defined by the prescription of a hypoglycemic drug for ≥28 d within 2 y before the date of screening or FPG ≥126 mg/dl at screening.	TB were defined as ICD-9-CM code 010–018 in the patient’s medical record plus prescription of anti-TB treatment for ≥90 d (including inpatient and outpatient services).	FPG, mg/dl (≤130, > 130)	Adjusted HR (Ref: No DM): FPG ≤130 mg/dl: 0.69 (0.35–1.36); FPG > 130 mg/dl: 2.21 (1.63–2.99)	Adjusted for age, sex, smoking status, alcohol use, betel nut use, education level, marital status, BMI, malignancy, pneumoconiosis, steroid use, ESRD, and frequency of outpatient visits.
Leung 2008	Active TB	Patients were recruited who enrolled at the 18 Elderly Health Service centers in Hongkong. DM was diagnosed, mainly by a FPG level of 7.0 mmol/liter or higher, together with confirmatory symptoms and/or blood/plasma glucose determinations.	The diagnosis of and clinical information on all identified TB cases were verified by reviewing medical records retrieved from chest clinics and other relevant sources, as well as the public health records of the TB and Chest Service. An active case of TB was defined as disease proven by isolation of Mycobacterium TB or, in the absence of bacteriologic confirmation, disease diagnose don clinical, radiologic, and/or histologic grounds together with an appropriate response to anti-TB treatment.	HbA1c (< 7%, ≥7%)	Crude RR (Ref: no DM): HbA1c < 7%: 0.64 (0.35, 1.16); HbA1c ≥ 7%: 1.97 (1.51, 2.57)	
Qiu 2017	TB	The data used were from Shanghai community-based DM management system (SCDMS), a DM register operated by the Shanghai Municipal Centers for Disease Control and Prevention (Shanghai-CDC). The diagnosis of DM must be verified by physicians in Community Health Centers (CHCs) using 1999 World Health Organization (WHO) criteria.	All TB diagnoses were confirmed by laboratory-based diagnostic tests using the China National TB Diagnostic Guidelines, including acid-fast bacilli (AFB) smear and culture test, purified protein derivative (PPD) skin test and serological test for *Mycobacterium TB infection* (Mtb).	Initial fasting glucose (mmol/L); Fasting glucose change (estimated by subtracting the initial values from the means of follow-up) (mmol/L)	Adjusted HR: initial fasting glucose (mmol/L): men: 1.21 (1.15,1.27); women: 1.27 (1.18,1.37); fasting glucose change (mmol/L): men: 1.17 (1.11,1.24); women: 1.27 (1.16,1.40) < 0.01	Not found
Case-control study						
Khalil 2016	Active TB	Patients were considered to be diabetic if they had a previous history of DM and were receiving antidiabetic therapy or were later found to have fasting plasma glucose ≥7.0 mmol/l (126 mg/dl). Or with a glucose tolerance test, two hours after the oral dose of plasma glucose 11.1 mmol/l (200 mg dl). Glycated hemoglobin (HbA1c) of greater than 6.5% is another method of diagnosis, also random blood sugar of greater than ≥11.1 mmol/l (200 mg/dl) in association with typical symptoms.	Patients were considered TB if at least two initial sputum smears positive for AFB (acid fast bacilli); or one sputum examination positive for AFB & radiographic abnormalities consistent with active pulmonary TB; or one sputum positive for AFB & culture positive for M. TB, and considered a new case if patient has never had treatment for TB or who has taken anti-TB drugs for less than one month.	Fasting blood sugar (mean); post prandial blood sugar (mean), HbA1c (mean)		
Leal 2019	TB	All the diabetics seen at the 30 municipal health units of Vitória, ES, Brazil were recruited.	Patients who were had a history of TB diagnosis and were notified at the Sistema de Informação de Agravos de Notificação (SINAN – Information System for Notifiable Diseases).	FBG (mean), PPG (mean), HbA1c (mean)		
Leegaard 2011	Active TB	DM was defined as previous in- or outpatient hospital contact involving DM, any use of oral anti-DM drugs or insulin, at least one visit to a chiropodist for DM foot care, at least five glucose-related services in general practice in 1 year, or at least two glucose-related services each year during 5 subsequent years.	Cases of active TB either had *Mycobacterium tuberculosis* complex (except *M. bovis* Bacillus Calmette-Guérin) isolated from a clinical specimen, or had *M. tuberculosis* DNA detected by PCR analysis, acid-fast bacilli demonstrated by direct microscopy, granuloma detected by histology, or had signs, symptoms, and/or radiological findings consistent with active TB in any site.	HbA1c, % (< 7.0, 7.0–7.9, ≥8.0)	Adjusted OR (Ref: No DM): < 7.0: 0.91 (0.51–1.63); 7.0–7.9: 1.05 (0.41–2.66); ≥8.0: 1.19 (0.61–2.30)	Adjusted for level of comorbidity, alcoholism-related disorders, marital status, number of children under the age of 15, and degree of urbanization.
Marupuru 2017	TB	Patients were recruited from a tertiary care hospital in South-India (Kasturba Hospital (KH), Manipal). Data were obtained from were obtained from the Medical Records Department of the hospital.	Subjects were identified based on ICD-10 coding for disease classification (TB: A15–A19).	FBS, mg/dl (median, < 70, 70–100, > 100); HbA1c, %, (median, < 7.0, 7.0–8.0, > 8.0)	Crude RR (Ref: HbA1c ≥7%): HbA1c < 7%: 0.52 (0.29, 0.93)	
Widjaja 2018	TB	Patients were recruited from MurniTeguh Memorial Hospitals in Medan, Indonesia.	Only patients diagnosed with diabetes and who gave signed informed consent were admitted to the study.	Blood glucose (mean), HbA1c (mean)		
Cross–sectional study						
Almeida-Junior 2016	TB	Patients were recruited from the Instituto Brasileiro para a Investigação da Tuberculose (IBIT, Brazilian Institute for TB investigation). The presence of DM was defined in accordance with American DM Association (ADA) guidelines as 2-h glucose ≥11.1 mmol/L, HbA1c ≥ 6.5% or fasting plasma glucose ≥7.0 mmol/L.	Diagnosis of TB at IBIT follows the guidelines of the Brazilian Society of Pulmonology and Tisiology, which is similar to WHO recommendations.	HbA1c, % (< 7, ≥7%); fasting glucose, OGTT	Adjusted OR: HbA1c: 1.40 (1.25–1.56); fasting glucose: 1.01 (1.004–1.01); OGTT: 1.01 (1.002–1.014) (for increases of 1 unit in plasma values of HbA1c, fasting glucose or OGTT glycaemia (after log10 transformation))	Adjusted for age, gender and BMI
Berkowitz 2018	Active TB	Participants were recruited from a DM clinic, where their diagnosis had been previously made.	TB screening and diagnoses were conducted using the national TB management guidelines. Participants were classified as having subclinical TB if diagnosed with active TB but with an absence of any clinical symptoms. An active TB case was defined as persons who tested positive for *M. tuberculosis* by either GeneXpert, smear microscopy, or TB culture in the presence or absence of clinical symptoms.	HbA1c, % (< 7, > 7%); FPG	Crude RR (Ref: HbA1c < 7%): HbA1c ≥ 7%: 3.07 (0.37–22.60)	
Chan 2019	Active TB	Patients were recruited if he/she registered in the National DM Registry.	Three sputum samples for AFB stain were obtained from each subject who presented with cough. Sputum smear positive PTB was defined as having at least one sputum sample positive for AFB, a CXR result consistent with typical PTB (consolidation/cavitations of an upper lung zone) and/or having symptoms of PTB (cough for > 2 weeks, weight loss, night sweats or fever for > 4 weeks). Subjects with sputum smear negative for AFB or having other symptoms of PTB with an abnormal CXR with typical findings of active PTB were referred to a chest physician to exclude smear negative PTB. Chest physicians evaluated the subjects with either a CT scan of the thorax and/ or bronchoscopy with washings for AFB. Subjects were then classified as being either sputum smear positive or smear negative PTB.	HbA1c (mean)	Crude OR: 1.30 (95% CI: 1.01–1.76)	
Hensel 2016	Latent TB	Patients were refugees seen at a health clinic in Atlanta, GA, USA. Patients with HbA1c < 6.5% with a previous diagnosis of DM indicated in their medical chart were also defined as DM.	Patients were considered to have LTBI if the QFT results were positive and chest radiographs were negative.	HbA1c, % (median); random blood glucose, mg/dl (median)	Crude OR (95%CI): DM status (Ref: No DM): Pre-DM: 1.83 (1.30–2.58); DM: 2.19 (1.22–3.94); Adjusted OR (95%CI): DM status (Ref: No DM): Pre-DM: 1.65 (1.13–2.39); DM: 2.27 (1.15–4.48)	Adjusted for age, sex, BMI, TB incidence in country of origin, smoking status, and vitamin D level.
Kumpatla 2013	TB	Patients attending the hospital and suspected of having DM are screened using the 2 h 75 g oral glucose tolerance test. The diagnosis of DM is based on previous DM history or on the WHO’s criteria for the classification of glucose intolerance.	patients with cough for ⩾2 weeks or any suspicion of active pulmonary TB (PTB) or extra-pulmonary TB were categorized as having presumptive TB and were further investigated to confirm the disease. Two same-day sputum specimens from presumptive TB patients were collected in the DM clinic and transported to the government-run microscopy center for sputum smear microscopy by Ziehl-Neelsen staining. Patients with negative sputum smears or extra-pulmonary TB suspects underwent appropriate investigations such as chest radiography to confirm TB.	fasting and postprandial glucose, mg/dl (mean); HbA1c, % (< 7%, 7–8.9%, ≥9%)		
Martinez-Aguilar 2015	Latent TB	Subjects with a medical history of DM receiving hypoglycemic drugs and/or insulin treatment at IMSS primary healthcare services of Durango City, Durango, Guadalupe and Zacatecas, Zacatecas (cities located in the central region of Northern Mexico) were randomly selected and recruited.	Subjects with a positive TST but with no evidence of active TB were considered as having LTBI.	Fasting glucose, mg/dL (median); HbA1c, % (median, ≤7, > 7%)	Adjusted OR (Ref: HbA1c ≤7%): HbA1c > 7%: 2.52 (1.10–8.25)	Adjusted for age and gender.
Sanchez-Jimenez 2018	Active TB	Patients were recruited from the National Institute of Respiratory Diseases (INER) “Ismael Cosío Villegas”; DM were confirmed by the clinical history, glucose tolerance test, fasting glucose levels ≥126 mg/dl, and by HbA1c ≥ 6.5%.	Pulmonary TB diagnosis was based on clinical history, physical examination, chest X-rays, and positive Ziehl-Neelsen test in sputum.	The fasting glucose (median), HbA1c (median)		
Webb 2009	Active TB	All children and adolescents (0–21 years) with documented type I DM who were routinely assessed during the study period at the two hospitals were eligible. Type I DM was considered present if a diagnosis was previously made by a pediatric endocrinologist.	A diagnosis of probable pulmonary TB disease was made when all three of the following criteria were met: 1) CXR changes consistent with TB; 2) clinical features of TB disease: respiratory symptoms (cough > 2 weeks, hemoptysis, dyspnea) and constitutional symptoms (fever, night sweat, fatigue or weight loss); and 3) TST ⩾10 mm.	HbA1c (mean)	Crude hazard ratio (95%CI): 1.39 (1.18–1.63) for per unit increase in HbA1c at diagnosis with TB.	

Abbreviation: DM: Diabetes mellitus, FBG: Fasting blood glucose, FPG: Fasting plasma glucose, HbA1c: Glycated hemoglobin A1c, HR: Hazard ratio, LTBI: Latent tuberculosis infection, OGTT: Oral glucose tolerance test, OR: Odds ratio, PPG: Post prandial blood glucose, TB: Tuberculosis, RR: Relative risk

Results of quality assessment are listed in the appendix. The ascertainment of DM was mostly based on medical record in seven studies, and laboratory testing in one study. Similarly, the diagnosis of TB was based on medical record in seven studies, laboratory testing in one study, and self-reported in one study.

### Glycated haemoglobin A1c

**Fig. 2:**
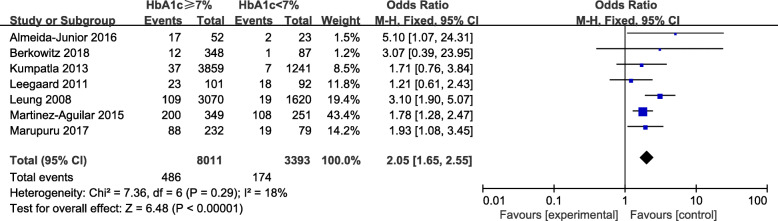
Forest plot of observational studies on poorly controlled diabetes mellitus and tuberculosis infection. Abbreviations: CI: confidence interval, HbA1c: glycated haemoglobin A1c, M-H: Mantel-Haenszel

**Fig. 3:**
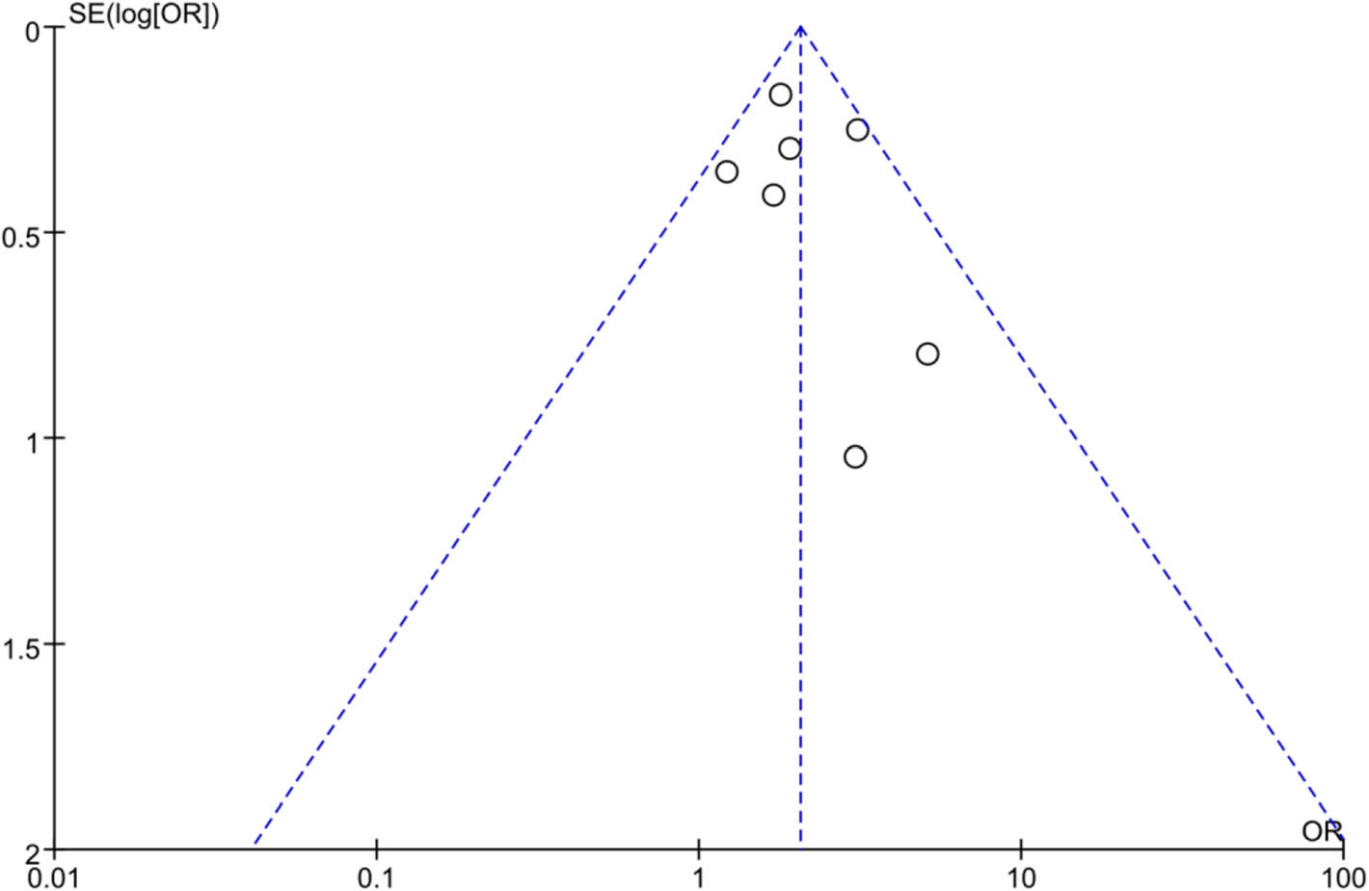
Funnel plot of observational studies on poorly controlled diabetes mellitus and tuberculosis infection

There were seven studies involved 20,857 participants to explore the relationship between glycemic control and TB [10, 11, 14, 17–20]. We conducted a meta-analysis to investigate the effect of poorly controlled glucose (HbA1c > 7.0%) on the prevalence of tuberculosis, and found that the pooled odds ratio of prevalent TB increased 2.05-fold (95%CI: 1.65–2.55) compared to good glycemic control (HbA1c < 7.0%) (Fig. 2). The crude OR from the observational studies revealed no substantial statistical heterogeneity (I^2^ = 18%, *P* = 0.29). Funnel plot was shown in Fig. 3. In the sensitivity analysis, we excluded the study (Leung 2008), which focused on the elderly, and found consistent results (Fig. S1).

**Fig. 4:**
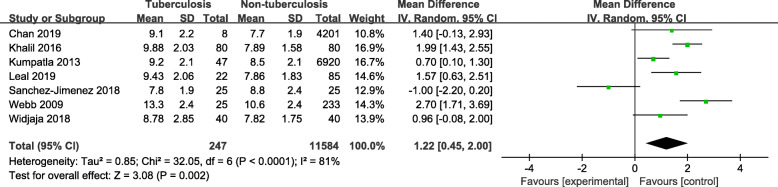
Forest plot of observational studies on glycated haemoglobin A1c and tuberculosis infection. Abbreviations: CI: confidence interval, IV: inverse variance

Table 3 exhibits the distribution of HbA1c concentrations in DM-TB patients and DM-only patients. Ten studies were included, and an increasing trend between non-DM and DM patients was observed in regard to their mean or median HbA1c concentrations [9, 14, 19–26]. Furthermore, there was a statistically significant difference in HbA1c concentrations between the two groups, and the mean of HbA1c was higher in the DM-TB than the DM-only group (*P* = 0.002, Fig. 4). Effect estimates were heterogeneous within studies (I^2^ = 81%, *P* <  0.0001). Then we restricted our study to the case-control studies, and observed consistent results. The funnel plot of the mean difference from three case-control studies revealed no statistical evidence of substantial heterogeneity (I^2^ = 34%, *P* = 0.22, Fig. S2).

**Table 3:** Association between glycated hemoglobin A1c concentrations and the prevalence of TB

Study	Type of study	Populations	TB			Non-TB			*P*
			Exposure	n	Mean ± SD/Median [IQR]	Exposure	n	Mean ± SD/Median [IQR]	
Chan 2019	A cross–sectional study	DM (n = 4209)	HbA1c (%)	8	Mean ± SD: 9.1 ± 2.2	HbA1c (%)	4201	Mean ± SD: 7.7 ± 1.9	Not shown
Khalil 2016	Case-control study	DM (n = 80)	HbA1c (%)	80	Mean ± SD: 9.88 ± 2.03	HbA1c (%)	80	Mean ± SD: 7.89 ± 1.58	< 0.01
Kumpatla 2013	A cross–sectional study	DM (n = 6967)	HbA1c (%)	47	Mean ± SD: 9.2 ± 2.1	HbA1c (%)	6920	Mean ± SD: 8.5 ± 2.1	0.03
Leal 2019	Case-control study	DM (n = 135)	HbA1c (%)	22	Mean ± SD: 9.43 ± 2.06	HbA1c (%)	85	Mean ± SD: 7.86 ± 1.83	0.002
Sanchez-Jimenez 2018	A cross–sectional study	DM (n = 50)	HbA1c (%)	25	Mean ± SD: 7.8 ± 1.9	HbA1c (%)	25	Mean ± SD: 8.8 ± 2.4	Not shown
Webb 2009	A cross–sectional study	DM (n = 258)	HbA1c (%)	25	Mean ± SD: 13.3 ± 2.4	HbA1c (%)	233	Mean ± SD: 10.6 ± 2.4	0.001
Widjaja 2018	Case-control study	DM (n = 80)	HbA1c (%)	40	Mean ± SD: 8.78 ± 2.85	HbA1c (%)	40	Mean ± SD: 7.82 ± 1.75	0.19
Hensel 2016	A cross–sectional study	Non-DM (n = 406), Pre-DM (n = 235), DM (n = 54)	HbA1c (%)	221	Median [IQR]: 5.7 [5.4–6.0]	HbA1c (%)	473	Median [IQR]: 5.5 [5.3–5.8]	< 0.01
Martinez-Aguilar 2015	A cross–sectional study	DM (n = 600)	HbA1c (%)	308	Median [IQR]: 7.5 [6.5–8.5]	HbA1c (%)	292	Median [IQR]:7.6 [6.8–8.7]	0.21
Marupuru 2017	Case–control study	DM (n = 451)	HbA1c (%)	152	Median [IQR]: 9.3 [7.5–11.6]	HbA1c (%)	299	Median [IQR]: 7.9 [6.8–10.7]	Not shown

Abbreviation: DM: Diabetes mellitus, HbA1c: glycated hemoglobin A1c, IQR: interquartile range, SD: standard deviation, TB: Tuberculosis

### Fasting plasma glucose

**Fig. 5:**
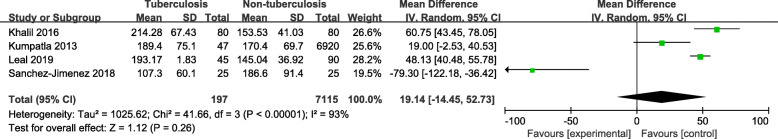
Forest plot of observational studies on fasting plasma glucose and tuberculosis infection. Abbreviations: CI: confidence interval, IV: inverse variance

Table 4 lists the distribution of FPG concentrations in TB-DM group and DM group. The seven studies involved 656 TB-DM patients and 8127 DM patient from five countries [14, 18–20, 22–24]. In statistical analysis, we focused on the fasting blood glucose index to explore the distribution of fasting blood glucose concentrations in TB-DM group and DM group. Due to the small sample sizes and the skewed data, means and medians can be very different from each other, so only means were used for meta-analyses. Therefore, we further conducted analysis in four articles for meta-analysis to explore the difference of mean FPG concentrations in the two groups (Fig. 5). The statistical analysis showed similar levels of FPG between TB-DM and DM-only groups (*P* = 0.26). There was significant heterogeneity of effect estimates, and 93% of the total variance among those studies. We further conducted analysis in case-control studies. The results showed no substantial statistical heterogeneity (I^2^ = 42%, *P* = 0.19), and observed significant difference in the two groups of TB-DM and DM-only patients (Fig. S3).

**Table 4:** Association between FPG and the prevalence of TB

Study	Type of study	Populations	TB			Non-TB			*P*
			Exposure	n	Mean ± SD/Median [IQR]	Exposure	n	Mean ± SD/Median [IQR]	
Leal 2019	Case-control study	DM (n = 135)	FPG (mg/dL)	31	Mean ± SD: 193.17 ± 1.83	FPG (mg/dL)	90	Mean ± SD: 145.04 ± 36.92	0.001
Khalil 2016	Case-control study	DM (n = 80)	FPG (mg/dL)	80	Mean ± SD: 214.28 ± 67.43	FPG (mg/dL)	80	Mean ± SD: 153.53 ± 41.03	< 0.01
Kumpatla 2013	A cross–sectional study	DM (n = 6967)	FPG (mg/dL)	47	Mean ± SD: 189.4 ± 75.1	FPG (mg/dL)	6920	Mean ± SD: 170.4 ± 69.7	0.17
Sanchez-Jimenez 2018	A cross–sectional study	DM (n = 50)	FPG (mg/dL)	25	Mean ± SD: 107.3 ± 60.1	FPG (mg/dL)	25	Mean ± SD: 186.6 ± 91.4	< 0.0001
Berkowitz 2018	A cross–sectional study	DM (n = 440)	FPG (mmol/L)	13	Median [IQR]: 8.2 [6.3–10.4]	FPG (mmol/L)	421	Median [IQR]: 8.2 [6.1–11.7]	Not shown
Martinez-Aguilar 2015	A cross–sectional study	DM (n = 600)	FPG (mg/dL)	308	Median [IQR]: 164 [126.2–237.5]	FPG (mg/dL)	292	Median [IQR]: 169 [126.5–234.5]	0.81
Marupuru 2017	Case–control study	DM (n = 451)	FPG (mg/dL)	152	Median [IQR]: 178 [124–243]	FPG (mg/dL)	299	Median [IQR]: 163 [125–234]	Not shown

Abbreviation: CI: confidence interval, DM: Diabetes mellitus, FPG: fasting plasma glucose, IQR: interquartile range, OR: odd ratio, SD: standard deviation, TB: Tuberculosis

## Discussion

In the present study, we conducted a meta-analysis that included seventeen studies and revealed that uncontrolled high levels of blood glucose of diabetic patients may be associated with an increased prevalence of tuberculosis. It suggests that tuberculosis screening among uncontrolled diabetic patients is necessary.

As the great burden of TB globally, it is important to identify potential high-prevalence populations appropriate for TB screening. Several studies have demonstrated that DM patients are at a greater risk for active TB disease, and poor glycemic control could also exacerbate this risk [10–14, 27]. As reported, 422 million people currently have diabetes, and 1.6 million deaths are directly attributed to diabetes each year (World Health Organization, Diabetes, https://www.who.int/health-topics/diabetes#tab=tab_1). The overlap between the diabetes and tuberculosis epidemics could adversely affect global tuberculosis control efforts. Then we conducted a meta-analysis to explore the relationship between uncontrolled DM and TB prevalence. Assessing glycemic control in DM patients, however, is still a challenge. Several recent approaches include the use of self-monitoring of blood glucose and HbA1c [16]. HbA1c testing was recommended as a simple and convenient method for evaluating average glycemia level over the preceding several months. In this study, we found the pooled odds ratio of prevalent tuberculosis increased a 2.05-fold (95%CI: 1.65, 2.55) for the patients with HbA1c ≥7.0% compared to those with HbA1c concentration < 7.0%. We also observed higher HbA1c levels in the TB-DM group compared to DM-only patients.

Glycated hemoglobin A1c is the product of glucose binding to hemoglobin in red blood cells. It is a gold standard for evaluating blood glucose control. Since most of the hemoglobin of human body exists in the red blood cell, which has a biological half-life of approximately 3–4 months. Thus, HbA1c concentrations reflects the controlling status of blood glucose over a relatively long-term peroid [28]. According to the American Diabetes Association (ADA) and the Canadian Diabetes Association (CDA) guidelines, HbA1c was accepted as an approved indicator for the assessment of glycemic control state in DM patients [29, 30]. In the present study, after evaluating plasma glucose levels through laboratory tests, there was a statistically significant difference in the mean of HbA1c between the two groups (TB-DM vs. DM-only group), suggesting these diabetics with poor glucose control may also have increased susceptibility to TB infection. Furthermore, diabetic patients with an HbA1c > 7.0 (%) had a twofold higher prevalence of TB than those with an HbA1c < 7.0 (%). Results of two studies reported that chronic hyperglycemia with HbA1c values of > 7%, had a 2.52–3.07 risk of developing pulmonary TB when compared with a group with better glycemic control [18, 20]. There are several possible explanations for the discrepancy. First, different tuberculin-skin-test thresholds are proposed for different countries and risk groups, which may affect the prevalence of tuberculosis. Moreover, tuberculosis are chronic wasting diseases and closely related to external factors, such as age, gender, BMI, nutrition, and contact history with binding patients. These factors may cause different results among a large number of existing studies, which also suggests that it is necessary for us to adopt a unified diagnosis and treatment standard to explore the relationship between glucose control and tuberculosis prevalence in a larger sample size meta-analysis or original literature.

Fasting plasma glucose is measured after overnight fasting or not eating anything for at least eight hours, which is the preferred method of screening test for DM in primary care. In the study, we included four studies for meta-analysis and found no statistically significant differences of FPG level between the DM-only group and TB-DM group. That may be due to the substantial statistical heterogeneity among studies. We further conducted analysis restricted to the two case-control studies. The results show no publication bias, and observed a high concentration of FPG in the TB-DM group. Consistent with previous studies, the strong association between heightened values of FPG and the prevalence of TB was claimed [31, 32]. In a Taiwanese cohort with five years of follow-up among 120,000 participants, Lee et al. reported 7.5% of incident TB could be attributed to poor glycemic control in the study population [32]. In the future, more researches are recommended to strengthen and explore the relationship between DM control and TB in the field.

The mechanisms that may underlie this association between TB infection and uncontrolled DM remain uncertain. Dysglycemia in DM patients was postulated to damage their innate or adaptive immunity and could trigger a hyperinflammatory state [26, 33]. It was reported that the synthesis of cytokines in pulmonary TB with DM patients shown significant changes compared to pulmonary TB without DM, such as IL-2, IL-6, IL-17, TNF-α and IFN-γ [34, 35]. In addition, Sanchez-Jimenez et al. showed the plasmatic increment of IL-15 could be related to the inflammatory state characteristic among DM and TB patients [24]. Further, a hyperinflammatory state of diabetic patients may favour the re-activation of TB [36, 37]. Despite of the potential association, policy on screening for TB infection among diabetic patients is not adopted by global health organizations [38]. The results of our study may provide more evidence that cost-effectiveness screening for tuberculosis in uncontrolled DM patients is needed and should be a high priority.

This study had some limitations. First, in the study, we pooled raw data to explore the prevalence of TB in uncontrolled DM patient. We could not control for potential confounding, such as alcohol consumption, nutrition, smoking status, and history of Bacillus Calmette-Guerin vaccination, diabetes duration [39], although we stratified by participant’s age in the sensitivity analysis. Second, most of studies that we have included are cross-sectional or case-control design, and as such we were unable to determine the temporal relationship between tuberculosis and diabetes. It restricts the popularization and application in this study. Moreover, it should be noted that the relationship between diabetes glycemic control and pulmonary tuberculosis may be complex or nonlinear, and more relevant studies are needed to explore. Third, the differences in the characteristics of the diabetes group and the control group, and different clinical evaluation markers for blood glucose control also bring difficulties to meta-analysis. There is need for well-designed prospective and larger studies in the future.

## Conclusion

In conclusion, we revealed that uncontrolled high levels of blood glucose of diabetic patients might be especially vulnerable to developing tuberculosis. Our findings suggested that tuberculosis controls strategies should consider jointly targeting diabetes patients with poorly controlled glucose. We also recommended further studies for more thorough understanding of the relationship that could deliver a clear public health message.

## Supplementary Information

**Additional file 1.** Search strategy for the systematic review.

**Additional file 2 Table S1**. Results of quality assessment for 9 studies.

**Additional file 3 Fig. S1**. Forest plot of observational studies on poorly controlled DM and tuberculosis infection excluded the study with the elderly. **Fig. S2**. Forest plot of observational studies on glycated haemoglobin A1c concentrations and tuberculosis infection with restricting to the case-control studies. **Fig. S3**. Forest plot of observational studies on fasting plasma glucose concentrations and tuberculosis infection with restricting to the case-control studies.

## Data Availability

The data used in the analyses described in this study are available from the author (QL, liuqihust2016@126.com) on reasonable request.

## Abbreviations

HbA1c: hemoglobin A1c
CI: confidence interval
TB: tuberculosis
DM: diabetes mellitus
HR: hazard ratio
FPG: fasting plasma glucose
IQR: inter-quartile range
RR: relative risk
SD: standard deviation
