# Supplementary material for: The association of glycemic level and prevalence of tuberculosis: a meta-analysis
Source: BMC Endocr Disord. 2021 Jun 16;21:123. doi: 10.1186/s12902-021-00779-6 (PMC8207612; doi:10.1186/s12902-021-00779-6)
Supplement: Supplementary file 1 — Additional file 1. Search strategy for the systematic review. [file 12902_2021_779_MOESM1_ESM.docx]

**The association of glycemic level and prevalence of tuberculosis: a meta-analysis**

**Appendix 1. Search strategy for the systematic review**

We searched PubMed, EMBASE and Web of Science through 14 December 2019.

**1. Search strategy in PubMed**

Search terms

#1 ("diabetes" OR "mellitus" OR "diabetes mellitus" OR "DM" OR "diabetes mellitus" [MeSH]) 707,737

#2 ("Glycated Hemoglobin A" OR "hemoglobin A1c" OR "HbA1c" OR "fasting plasma glucose" OR "FPG" OR "glycemic control" OR "glyc$" OR "Blood Glucose" [MeSH] OR "Glycated Hemoglobin A"[Mesh])) 206,871

#3 ("Tuberculosis" OR "TB" OR "Mycobacterium" OR "Tuberculosis"[ MeSH]) 308,487

#4 #1 AND #2 AND #3 293

**2. Search strategy in EMBASE**

Search terms

#1 'diabetes mellitus'/exp OR 'diabetes' OR 'mellitus' OR 'diabetes mellitus' OR

'DM' 1,375,084

#2 'glycated hemoglobin '/exp OR 'hemoglobin A1c'/exp OR 'glycemic control'/exp OR 'glucose blood level'/exp OR 'glycated hemoglobin' OR 'hemoglobin A1c' OR 'HbA1c' OR 'fasting plasma glucose' OR 'FPG' OR 'glycemic control' OR 'glucose blood level' 346,006

#3 'tuberculosis'/exp OR 'tuberculosis' OR 'mycobacterium'/exp OR 'mycobacterium' OR 'TB' 316,221

#4 #1 AND #2 AND #3 964

**3. Search strategy in Web of Science**

#1 TS=(Tuberculosis OR TB OR Mycobacterium) 471,783

#2 TS=(Glycated Hemoglobin A OR hemoglobin A1c OR HbA1c OR fasting plasma glucose OR FPG OR glycemic control OR glyc* OR Blood Glucose) 2,534,013

#3 TS=(diabetes OR mellitus OR diabetes mellitus OR DM) 1,156,241

#4 #1 AND #2 AND #3 995
