# Supplementary material for: The association of glycemic level and prevalence of tuberculosis: a meta-analysis
Source: BMC Endocr Disord. 2021 Jun 16;21:123. doi: 10.1186/s12902-021-00779-6 (PMC8207612; doi:10.1186/s12902-021-00779-6)
Supplement: Supplementary file 2 — Additional file 2 Table S1. Results of quality assessment for 9 studies. [file 12902_2021_779_MOESM2_ESM.docx]

**The association of glycemic level and prevalence of tuberculosis: a meta-analysis**

**Table S1**. Results of quality assessment for 9 studies.

| Study | Type of study | Location | Age | Period | Diagnosis of diabetes | Diagnosis of tuberculosis | Study Quality |
| --- | --- | --- | --- | --- | --- | --- | --- |
| Golub 2019 | Cohort study | Korea | Adults | 2001-2011 | Medical records | Medical records | S★★★★ C★★ O★★Overall 8 |
| Lee 2016 | Cohort study | Taiwan | ≥ 30 years | 2005-2012 | Medical records | Medical records | S★★★ C★★ O★★Overall 7 |
| Leung 2008 | Cohort study | China | ≥ 65 years | 2000-2005 | Medical records | Medical records | S★★★ C★★ O★★Overall 7 |
| Qiu 2017 | Cohort study | China | ≥18 years | 2004-2014 | Medical records | Medical records | S★★★★ C★O★★Overall 8 |
| Khalil 2016 | Case-control study | Egypt | All populations | 2014-2015 | Laboratory testing | Laboratory testing | S★★★★E★Overall 5 |
| Leal 2019 | Case-control study | Brazil | ≥18 years | 2007-2013 | Medical records | Self-reported？ | S★★★ C★E★Overall 5 |
| Leegaard 2011 | Case-control study | Denmark | Adults | 1980-2008 | Medical records | Medical records | S★★ ★C★★E★Overall 6 |
| Marupuru 2017 | Case-control study | India | ≥40 years | 2015-2016 | Medical records | Medical records | S★★★ E★Overall 4 |
| Widjaja 2018 | Case-control study | Indonesia | 36-86 years | 2017-2017 | Laboratory testing | Medical records | S★ E★★Overall 3 |
