# Supplementary material for: The association of glycemic level and prevalence of tuberculosis: a meta-analysis
Source: BMC Endocr Disord. 2021 Jun 16;21:123. doi: 10.1186/s12902-021-00779-6 (PMC8207612; doi:10.1186/s12902-021-00779-6)

**The association of glycemic level and prevalence of tuberculosis: a meta-analysis**

Figure S1. Forest plot of observational studies on poorly controlled DM and tuberculosis infection excluded the study with the elderly.

Figure S2. Forest plot of observational studies on glycated haemoglobin A1c concentrations and tuberculosis infection with restricting to the case-control studies.

Figure S3. Forest plot of observational studies on fasting plasma glucose concentrations and tuberculosis infection with restricting to the case-control studies.

Figure S1. Forest plot of observational studies on poorly controlled DM and tuberculosis infection excluded the study with the elderly.


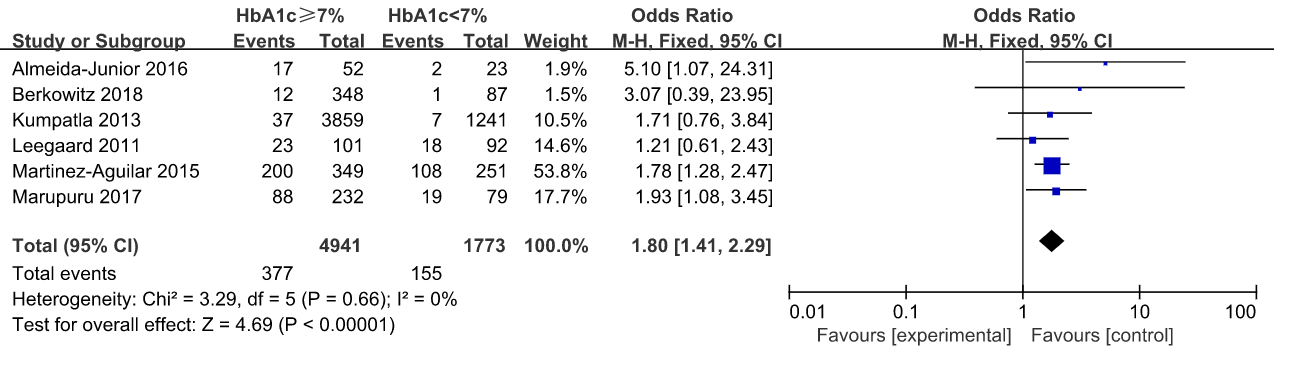


Abbreviations: DM: diabetes mellitus, HbA1c: glycated haemoglobin A1c, CI: confidence interval.

Figure S2. Forest plot of observational studies on glycated haemoglobin A1c concentrations and tuberculosis infection with restricting to the case-control studies.


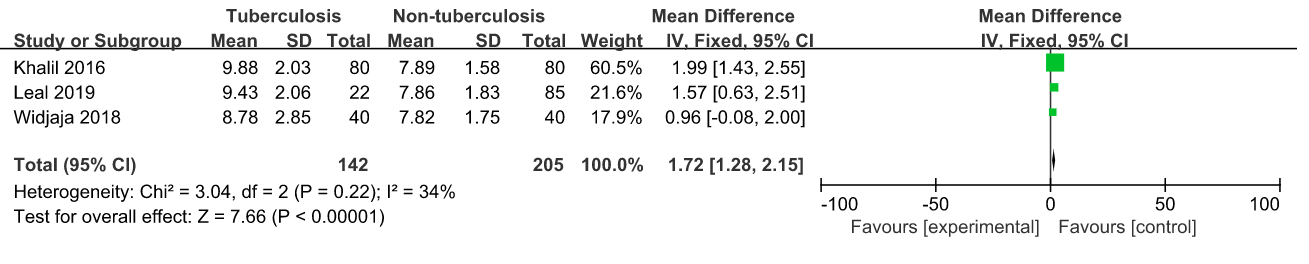


Abbreviations: CI: confidence interval.

Figure S3. Forest plot of observational studies on fasting plasma glucose concentrations and tuberculosis infection with restricting to the case-control studies.


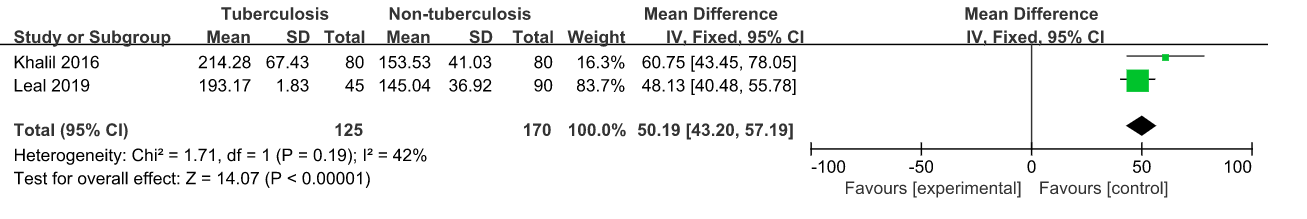

Supplement: Supplementary file 3 — Additional file 3 Fig. S1. Forest plot of observational studies on poorly controlled DM and tuberculosis infection excluded the study with the elderly. Fig. S2. Forest plot of observational studies on glycated haemoglobin A1c concentrations and tuberculosis infection with restricting to the case-control studies. Fig. S3. Forest plot of observational studies on fasting plasma glucose concentrations and tuberculosis infection with restricting to the case-control studies. [file 12902_2021_779_MOESM3_ESM.docx]
